# To Correspondents

**Published:** 1860-07

**Authors:** 


TO CORRESPONDENTS.—Communications have been received from Drs. Bell, Armor, and Don José de la Luz Hernandez.

